# Nonaqueous Electrolyte Rechargeable Manganese Batteries with Potassium Manganese Hexacyanoferrate Cathodes

**DOI:** 10.1002/advs.202500132

**Published:** 2025-03-28

**Authors:** Jangwook Pyun, Hyeonjun Lee, Seunghyeop Baek, Sangki Lee, Hyeju Kwon, Hyeongseok Lee, Chung‐Yul Yoo, Munseok S. Chae

**Affiliations:** ^1^ Department of Nanotechnology Engineering Pukyong National University Busan 48547 Republic of Korea; ^2^ Department of Energy Systems Research Ajou University Yeongtong‐gu Suwon 16499 Republic of Korea

**Keywords:** manganese batteries, manganese hexacyanoferrate, nonaqueous battery, organic electrolyte

## Abstract

Manganese batteries garnered significant attention as sustainable and cost‐effective alternatives to lithium‐ion batteries. For the first time, manganese batteries are demonstrated using a manganese hexacyanoferrate cathode and organic electrolyte solution, specifically saturated Mn(ClO₄)₂ in acetonitrile. The manganese hexacyanoferrate cathode exhibits an average operating voltage of 1.7 V and a discharge capacity of 73.4 mAh g^−1^ at 0.1 A g^−1^, retaining 71.1% capacity after 1500 cycles at 0.2 A g^−1^. Diffusion pathways and barriers reveal efficient 3D Mn^2^⁺ ion diffusion pathways within the manganese hexacyanoferrate framework, with a low migration barrier of 0.514 eV. Despite the promising performance, surface analysis of the Mn metal anode reveals the formation of complex organic/inorganic SEI (solid electrolyte interphase) layers, including MnO_x_, MnCl_x_, and organic compounds, due to electrolyte decomposition. These findings highlight the critical importance of SEI layer control and electrolyte optimization for enhancing the durability and efficiency of organic electrolyte‐based manganese batteries. Manganese batteries are established as a viable next‐generation energy storage solution and provide a foundation for further advancements in organic electrolyte‐based battery systems.

## Graphical abstract

This study presents manganese batteries utilizing a MnHCF cathode using an organic electrolyte solution. The MnHCF demonstrates high performance and durability. Furthermore, the organic/inorganic SEI layers formed on the Mn metal anode are comprehensively analyzed.

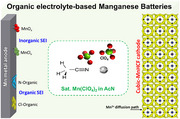

## 1 Introduction

Rechargeable batteries using aqueous electrolytes garnered significant attention as a sustainable alternative to conventional lithium‐ion batteries.^[^ ^1^ ^]^ This growing interest is driven by their enhanced safety, cost‐effectiveness, and environmentally friendly characteristics. The inherent safety of water‐based electrolytes, which dramatically reduces the risks of fire and explosion, makes these systems particularly appealing for large‐scale energy storage applications. Additionally, their minimal environmental footprint aligns closely with the global shift toward sustainable and green energy technologies.

Over the years, aqueous rechargeable batteries have primarily used metal anodes such as zinc,^[^ ^2^ ^]^ due to their high theoretical specific capacities and chemical stability. Despite these advantages, several challenges persist. The zinc‐based batteries, often regarded as the most advanced anode material in aqueous systems, face difficulties achieving high energy densities due to their relatively high redox potential (‐0.76 V vs SHE).^[^ ^3^ ^]^ Addressing these limitations is critical for advancing aqueous battery technology and realizing its full potential in pursuing safe, sustainable, and high‐performance energy storage solutions.

Aqueous Mn‐based batteries emerged as a highly promising candidate in advanced energy storage technologies.^[^ ^4^ ^]^ Their appeal lies in a combination of exceptional properties, including high theoretical gravimetric and volumetric capacities (976 mAh g^−1^ and 7250 mAh cm^−^^3^, respectively), the earth abundance and cost‐effectiveness of manganese as a raw material, and its low redox potential (−1.19 V vs SHE). This low redox potential of Mn metal enables higher operating voltages than those of Zn‐based battery systems, offering a significant advantage in performance. Furthermore, Mn‐based batteries can be safely assembled under ambient conditions, providing a streamlined and efficient fabrication process and enhancing their practicality.^[^ ^5^ ^]^

Despite these promising attributes, the advancement of manganese‐based storage host structures remains relatively underdeveloped. This limitation largely stems from the divalent charge state of manganese ions, which poses significant challenges in developing stable and efficient host materials like usual multivalent‐ion batteries. Overcoming these obstacles is crucial to unlocking the full potential of aqueous manganese‐based batteries and facilitating their adoption in sustainable, high‐performance energy storage technologies.

Despite recent advancements, the range of electrode materials investigated for reversible operation in Mn‐based electrolytes remains limited. To date, prominent examples include the Chevrel phase Mo₆S₈,^[^ ^6^ ^]^ alpha‐V₂O₅,^[^ ^6^ ^]^ Ag_0.11_V_2_O_5,_^[^ ^7^ ^]^ Ag_0.33_V_2_O_5,_^[^ ^4^, ^8^ ^]^ VO_2_,^[^ ^9^ ^]^ Ni‐based Prussian blue analogue,^[^ ^6^ ^]^ layered‐Al_0.1_V_2_O_5_·1.5H_2_O,^[^ ^10^ ^]^ Mn_0.18_V_2_O_5_·nH_2_O,^[^ ^11^ ^]^ and organic electrodes such as PTCDA^[^ ^4^, ^12^ ^]^ and coronene.^[^ ^4b^ ^]^ However, the discovery of new electrode materials for manganese‐ion batteries remains a critical area of research.

Another significant challenge lies in the choice of electrolytes. Manganese‐ion batteries typically use aqueous electrolytes, although their use often leads to the undesirable hydrogen evolution reaction (HER) due to the nature of the aqueous solution.^[^ ^13^ ^]^ Therefore, research on alternative electrolytes, such as organic (nonaqueous) or water‐organic hybrid electrolytes (wet‐organic systems), is essential. However, despite the critical importance of this issue, no substantial reports or progress, even at the fundamental research level, were reported. If these challenges related to both cathode materials and electrolytes are effectively addressed, manganese‐ion batteries have the potential to emerge as a highly promising next‐generation energy storage system, garnering significant attention for their advanced capabilities.

Therefore, we designed manganese‐ion batteries based on nonaqueous electrolytes and introduced a novel cathode material, manganese hexacyanoferrate (MnHCF).^[^ ^14^ ^]^ MnHCF is recognized as one of the most promising materials for multivalent‐ion battery systems. Similar Prussian blue‐type structures were extensively studied for their ability to host a variety of divalent and trivalent cations, including Mg^2^⁺,^[^ ^15^ ^]^ Zn^2^⁺,^[^ ^16^ ^]^ Ca^2^⁺,^[^ ^17^ ^]^ and Al^3^⁺.^[^ ^18^ ^]^ This versatility is attributed to their 3D open framework, which provides ample space to accommodate cations. Furthermore, MnHCF typically operates at a high voltage of ≈3.7 V (vs Li/Li⁺),^[^ ^19^ ^]^ making it an excellent candidate for high‐voltage energy storage systems. We used nonaqueous electrolyte solutions, acetonitrile (AcN) and propylene carbonate (PC), which demonstrated high coulombic efficiency in zinc‐based organic electrolytes.^[^ ^16^ ^]^

Here, we demonstrate, for the first time, nonaqueous manganese‐ion batteries using a MnHCF cathode and Mn(ClO_4_)_2_ in an acetonitrile‐based electrolyte. Basic electrochemical tests were conducted to characterize the electrodeposition of Mn metal. The MnHCF cathode exhibited good reversibility, delivering a capacity of ≈73 mAh g^−1^ at a current density of 100 mA g^−1^ and retaining 71.1% of its capacity after 1500 cycles at 0.2 A g^−1^ in a saturated Mn(ClO_4_)_2_ acetonitrile electrolyte solution. Furthermore, Mn diffusion pathways and energy barriers were calculated to elucidate the reaction mechanisms within the Prussian blue analog framework. Surface analysis of the Mn metal anode revealed the presence of decomposition products from the organic electrolyte, providing valuable insights into their impact on Mn battery performance. We present a preliminary investigation aimed at overcoming the limitations of conventional aqueous systems and advancing the development of high‐performance manganese‐ion batteries.

## 2 Results and Discussion

### 2.1 Electrochemical Mn Metal Plating in Organic‐Based Electrolyte Solutions

To select an organic solution, we referred to Zn‐based organic electrolyte systems. The standard electrode potential of Mn/Mn^2^⁺ is similar to that of Zn/Zn^2^⁺, and the Zn metal anode demonstrates ≈100% coulombic efficiency in organic electrolytes, particularly in PC and AcN.^[^ ^16^, ^20^ ^]^ Therefore, this study explores electrolyte systems based on organic solvents, specifically AcN and PC. For the electrolyte test, Pt electrode and Pt mash were composed as working and counter electrodes, respectively, and Mn metal was used as a reference.

**Figure 1:**
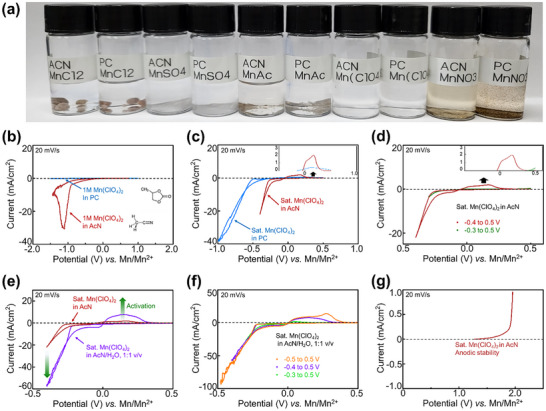
a) Images of dissolution test combinations of various Mn salts and acetonitrile (AcN) /propylene carbonate (PC) solutions, electrochemical Mn metal deposition dissolution test under pure organic electrolyte solution: b) 1 m and c) saturated Mn(ClO_4_)_2_ AcN or PC solutions, d) voltage range test, electrochemical Mn metal deposition dissolution test under wet‐organic electrolyte e) Saturated Mn(ClO_4_)_2_ in AcN/H_2_O mixed solutions, f) voltage range test, g) anodic stability of saturated Mn(ClO_4_)_2_ in AcN.

To evaluate the solubility of various manganese salts in organic solvents, salts were added to the solutions, as shown in **Figures** **1**a and S1 (Supporting Information). MnCl₂, MnSO₄, and Mn acetate did not dissolve into AcN or PC solution, while Mn(NO₃)₂ showed slight solubility but formed a brown precipitate. Among the salts tested, Mn(ClO₄)₂ exhibited the best solubility in organic solvents (AcN or PC). Electrochemical tests for manganese metal deposition were conducted using 1 m Mn(ClO₄)₂ in AcN or PC solutions. In 1 m Mn(ClO₄)₂ in PC, no current was observed, whereas, in AcN, only reduction current was detected without oxidation current in cyclic voltammopgtam (CV), suggesting that the observed reactions likely stem from electrolyte decomposition reactions rather than manganese deposition (Figure 1b). When the Mn(ClO₄)₂ was saturated, small oxidation peaks emerged, suggesting that at lower concentrations, high desolvation energy prevents manganese deposition. However, when saturated Mn(ClO₄)₂ was utilized in AcN or PC, the solvation numbers decreased and offered lower desolvation energy (Figure 1c). Manganese deposition tests at various voltages revealed that manganese did not deposit at −0.3 V but required −0.4 V to overcome desolvation energy and enable deposition in saturated Mn(ClO₄)₂ in AcN solution (Figure 1d). However, the deposition–dissolution efficiency was extremely low, prompting further experiments with wet organic electrolytes to understand the characteristics of the organic electrolyte. In a 1:1 v/v AcN/H₂O mixture, significantly enhanced reduction and oxidation currents were observed (Figure 1e). Although HER occurred with adding water, the lower desolvation energy facilitated manganese deposition. The wet‐organic electrolyte showed activated oxidation peaks within the lower reduction voltage range (Figure 1f), demonstrating the importance of organic electrolyte selection for achieving reversible manganese deposition and dissolution. Manganese ions form stronger solvation bonds with organic solvents than those with water, resulting in higher overpotentials required for manganese deposition.

The solution was oxidized to further assess the anodic stability of saturated Mn(ClO₄)₂ in Figure 1g. Stable performance was observed up to 1.5 V; however, decomposition of AcN occurred at 1.9 V.

**Figure 2:**
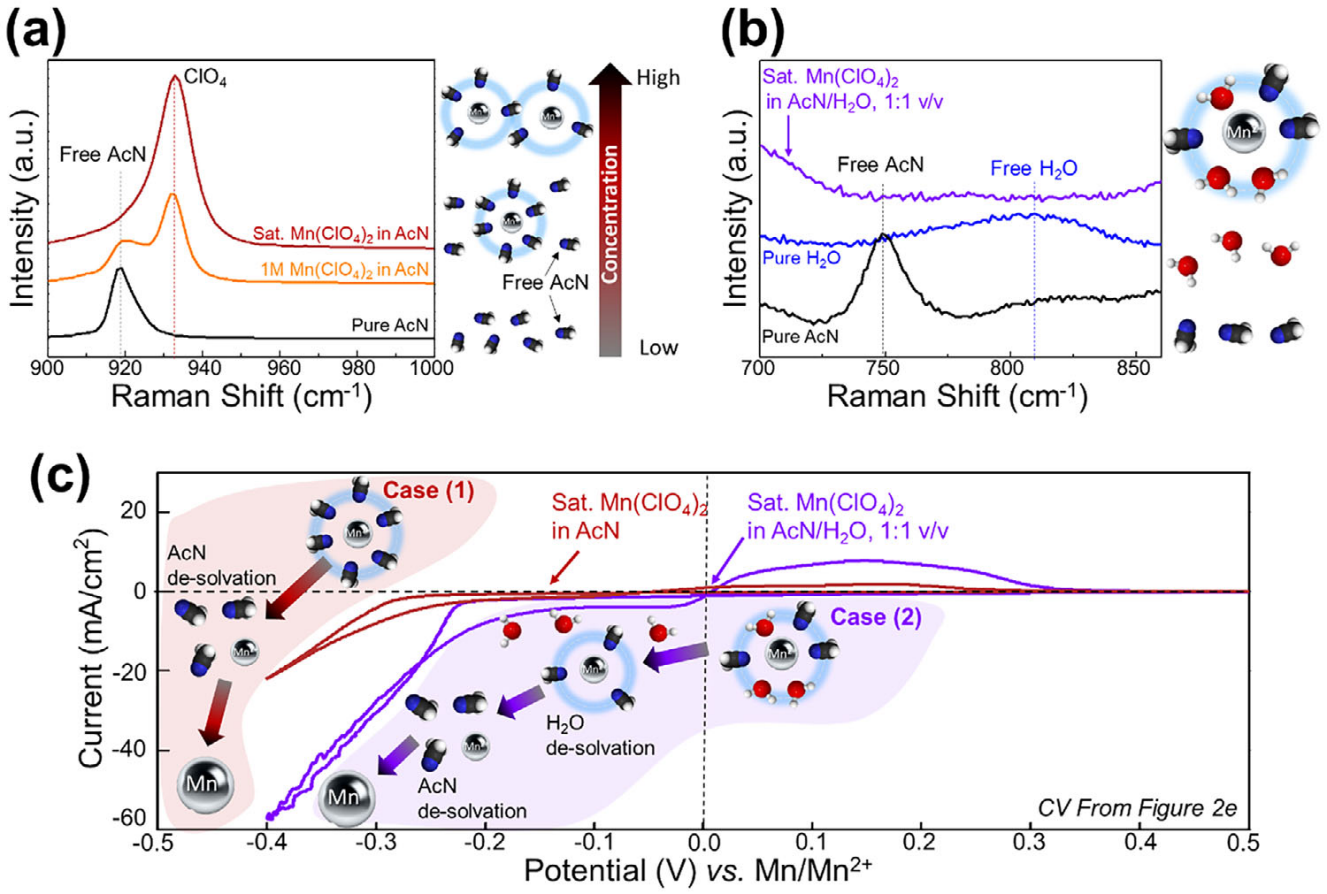
Raman spectra include: a) pure AcN and 1 m/saturated Mn(ClO_4_)_2_ in AcN solution, b) pure AcN/water and saturated Mn(ClO_4_)_2_ in AcN/H₂O solution, and c) a schematic illustration of the reaction mechanism between saturated Mn(ClO_4_)_2_ in AcN solution and AcN/H₂O solution.

To investigate the characteristics of the electrolytes, Raman spectra were measured and represented in **Figure** **2**a,b. For Mn(ClO₄)₂ in AcN, the pure AcN peak is observed at 918 cm⁻¹. As the concentration of Mn(ClO₄)₂ increases, the free AcN peak diminishes and disappears in the saturated solution, indicating that Mn(ClO₄)₂ salt is well solvated in AcN. Concurrently, the ClO₄⁻ peaks increase with the salt concentration (Figure 2a). Further analysis was conducted for saturated Mn(ClO₄)₂ in AcN/H₂O mixed solutions (wet organic electrolyte). This system demonstrates effective solvation of Mn(ClO₄)₂ salt by both water and AcN molecules. In the saturated electrolyte, the free AcN and H₂O peaks vanish, further confirming complete solvation by the solvent mixture (Figure 2b). Comparing the Mn deposition reactions in the two electrolytes, as shown in Figure 2c and Figure 1e, distinct differences were observed. The CV curves of saturated Mn(ClO₄)₂ in AcN exhibited significant polarization for Mn deposition. Contrastingly, the wet organic electrolyte (saturated Mn(ClO₄)₂ in AcN/H₂O) shows a two‐step deposition process. In the wet organic system, the first deposition step occurs in the voltage range of −0.2–0.0 V versus Mn/Mn^2^⁺, where water molecules are desolvated, allowing partial Mn metal deposition. The second step, below −0.25 V versus Mn/Mn^2^⁺, involves the desolvation of AcN molecules, resulting in Mn plating with a strong reduction current. In the AcN‐only electrolyte, Mn ions are primarily solvated by AcN molecules, requiring a more negative voltage below −0.3 V versus Mn/Mn^2^⁺ to overcome the higher AcN desolvation energy and enable Mn deposition.

These results indicate that the desolvation energy of water from Mn ions is lower than that of AcN, facilitating Mn deposition at less negative potentials in the wet organic electrolyte. This distinction underscores the critical influence of solvent composition on the electrochemical deposition behavior of Mn metal and highlights the potential of wet organic systems to reduce polarization and improve deposition efficiency. However, wet‐organic electrolytes still exhibit intrinsic challenges. To address this limitation and successfully demonstrate manganese‐ion batteries, we exclusively used organic electrolytes.

However, the AcN‐only electrolyte exhibits low deposition/dissolution efficiencies (7.35%), indicating that most of the reaction primarily results from AcN decomposition and/or the formation of irreversible Mn side products. Consequently, only 7.35% of Mn metal is deposited on the Pt electrode surface. In the case of a saturated Mn(ClO₄)₂ solution in an AcN/H₂O electrolyte, the deposition and dissolution efficiencies increase to 12.49%. However, this level remains insufficient for practical applications. For comparison, the coulombic efficiencies of Li, Na, and Zn metal anodes are ≈98%,^[^ ^21^ ^]^ 85%,^[^ ^22^ ^]^ and 99%,^[^ ^16^ ^]^ respectively. Additionally, the deposition/dissolution efficiency of Mn in an aqueous electrolyte is significantly higher, reaching ≈40% in a saturated Mn(ClO₄)₂ solution in pure water.^[^ ^4b^ ^]^

Compared to zinc, we initially expected high deposition efficiency in AcN and PC electrolytes due to the similar redox potentials of Zn/Zn^2^⁺ and Mn/Mn^2^⁺. However, the results did not meet our expectations. We guess this to the multiple oxidation states of manganese.

While zinc undergoes a simple two‐electron redox reaction (Zn ↔ Zn^2^⁺), manganese undergoes multiple oxidation state transitions (Mn ↔ Mn^2^⁺ ↔ Mn^3^⁺ ↔ Mn⁴⁺). These transitions alter the ion's charge density, making it more difficult for manganese to separate from the electrolyte, thereby affecting deposition behavior. Consequently, various side reactions are more likely to occur, leading to partial Mn deposition and the formation of MnO₂ or other irreversible byproducts.

Therefore, further research is needed to explore various combinations of manganese‐based organic electrolytes with different salts to improve efficiency and practicality.

### 2.2 MnHCF Cathode Synthesis and Characterizations

**Figure 3:**
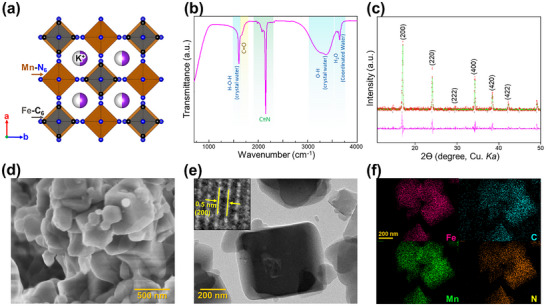
a) Crystal structure of MnHCF structure, as‐prepared MnHCF powder characterizations: b) FTIR spectra, c) Rietveld refinement from powder X‐ray diffraction data, d) SEM image, e) TEM image and high‐resolution lattice (inset), f) TEM‐EDX elemental mapping for Mn (green), Fe (pink), C (cyan), and N (orange).

To prepare cathode material, nanosized MnHCF was synthesized via a co‐precipitation method using K₃[Fe(CN)₆] and MnSO₄ solutions.^[^ ^23^ ^]^ The ideal MnHCF Prussian blue structure is shown in **Figure** **3**a. The successful synthesis of MnHCF was confirmed using Fourier‐transform infrared spectroscopy (Figure 3b), where a distinct CN stretching vibration was observed, indicating the presence of the MnHCF framework. Raman spectroscopy further corroborated this finding, with the CN signal visible (Figure S2, Supporting Information). Peaks associated with H₂O suggest incorporating crystal water into the structure, while the presence of a C ═ O peak implies the existence of structural defects typical of the normal cubic Prussian blue phase.^[^ ^24^ ^]^ Rietveld refinement of the powder X‐ray diffraction (XRD) data confirmed the formation of a cubic phase with an Fm3̅m space group and unit cell parameters of a = 10.492(1) Å (Figure 3c). Detailed crystallographic data are provided in Table S1. SEM images (Figure 3d) revealed particles with a cubic morphology and sizes ranging from 50–600 nm (Figure S2, Supporting Information). The charging effect observed in the SEM image indicates that MnHCF powder has low electron conductivity. TEM and high‐resolution lattice fringe imaging (Figure 3e and Figure S4, Supporting Information) showed lattice fringes corresponding to the d(200) plane, with a spacing of ≈0.5 nm. Elemental analysis was performed using energy‐dispersive X‐ray spectroscopy (EDS) coupled with TEM for elemental mapping and spectral analysis (Figure 3f, Figures S5,S6, Supporting Information). The results demonstrated a uniform distribution of Fe, Mn, C, and N throughout the MnHCF particles, with no evidence of impurity phases.

### 2.3 Electrochemical MnHCF/ /Mn Metal Cell

**Figure 4:**
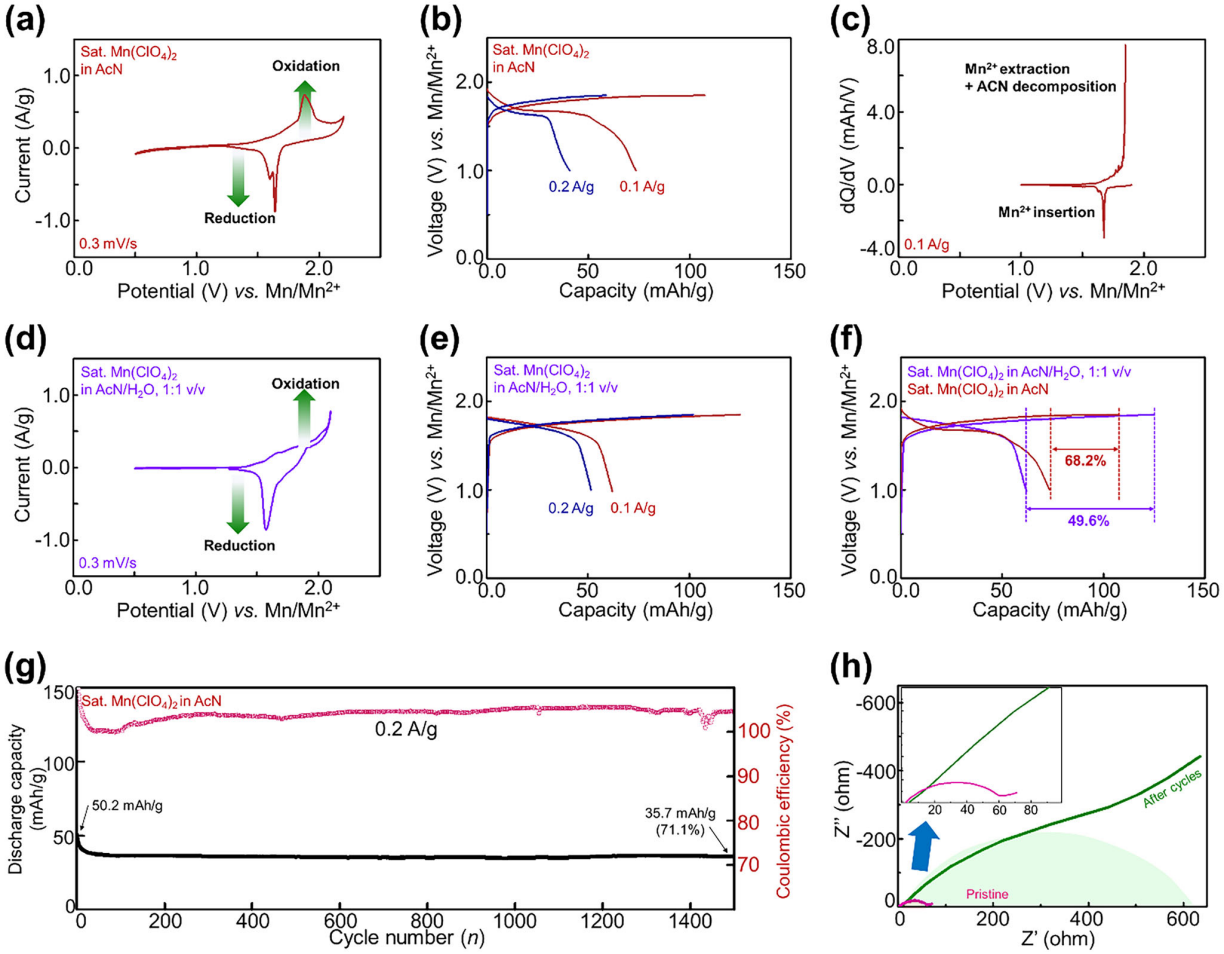
a) CV profile of MnHCF in a saturated Mn(ClO_4_)_2_ in AcN electrolyte; b) GDC profiles at current densities of 0.1 and 0.2 A g^−1^; c) dQ/dV curves derived from graph (b) at 0.1 A g^−1^; d) CV profile and e) GDC profiles of MnHCF at current densities of 0.1 and 0.2 A g^−1^ in a saturated Mn(ClO₄)₂ AcN/H₂O electrolyte; f) Comparison of charge‐discharge efficiencies in both electrolytes; g) long‐term cycling performance at 0.2 A g^−1^; h) impedance spectra before and after cycling.

While manganese‐ion batteries were previously demonstrated in aqueous‐based electrolytes, this study represents the first demonstration of manganese‐ion batteries using an organic electrolyte. The electrochemical cells employed in this work consist of a three‐electrode configuration, with a manganese metal foil as the anode, a manganese rod as the reference electrode, MnHCF as the cathode, and saturated Mn(ClO₄)₂ in AcN as the electrolyte. The CV of MnHCF (**Figure** **4**a) shows two reduction peaks at 1.64 and 1.60 V and one oxidation peak at 1.90 V versus Mn/Mn^2^⁺, within the voltage range of 0.5–2.2 V. These peaks correspond to the reversible Mn^2^⁺ ion intercalation/extraction reactions. The corresponding galvanostatic discharge–charge (GDC) profiles (Figure 4b) reveal an average operating voltage of 1.7 V, which is prominently high for this system. At a current density of 0.1 A g^−1^, MnHCF demonstrates a discharge capacity of 73.4 mAh g^−1^, which is competitive compared to that of other organic electrolyte‐based divalent ion battery systems employing cubic Prussian blue phases.^[^ ^15^, ^16^ ^]^ When the current density increases to 0.2 A g^−1^, the discharge capacity decreases to 40.8 mAh g^−1^, consistent with that of electrolyte decomposition as previously observed (Figure 2g). To further analyze the electrochemical behavior, dQ/dV curves were calculated at a current density of 0.1 A g^−1^ as shown in Figure 4c. The dQ/dV data aligns well with the CV results, with pronounced oxidation peaks observed. These peaks indicate simultaneous Mn extraction and AcN decomposition, contributing to the observed charge capacities.

We also conducted electrochemical tests using a saturated Mn(ClO₄)₂ solution in an AcN/H₂O mixed electrolyte. The CV profile of MnHCF (Figure 4d) exhibits a reduction peak at 1.57 V and an oxidation peak at ≈2.1 V versus Mn/Mn^2^⁺, within the voltage range of 0.5–2.1 V. The corresponding GCD profiles (Figure 4e) indicate that at a current density of 0.1 A g^−1^, MnHCF delivers a discharge capacity of 62.0 mAh g^−1^, which is lower than that observed in the AcN electrolyte‐based system. However, the rate performance in the mixed electrolyte system is superior to that of the AcN electrolyte. The primary issue in the saturated Mn(ClO₄)₂ AcN/H₂O mixed electrolyte system is the significant occurrence of electrolyte side reactions during charging. While the AcN electrolyte system exhibits a charge–discharge efficiency of 68.2%, the mixed electrolyte system shows a markedly lower efficiency of 49.6% (Figure 4f). This deterioration is likely attributed to the increased polarization of the redox potential, which hampers ion extraction during the charging process. Additionally, side reactions between water and the Mn metal anode may lead to the formation of a resistive surface layer, further contributing to the observed performance degradation. The cycling stability of MnHCF under saturated Mn(ClO₄)₂ in AcN as the electrolyte was evaluated at a current density of 0.2 A g^−1^ in Figure 4g. After 1500 cycles, MnHCF retained a capacity of 35.7 mAh g^−1^, corresponding to ≈71.1% retention of its initial capacity. However, unstable coulombic efficiencies were observed, likely due to electrolyte decomposition during oxidation. Electrochemical impedance spectroscopy was performed on the cell before and after 1500 cycles (Figure 4h). Initially, the internal resistance (R1​) was 2.94 Ω, although it increased to 4.57 Ω after cycling, indicating a degradation of mechanical contact and increased cell resistance. The charge transfer resistance rose significantly, from 59.1 Ω (R2) initially to 520.5 Ω (R2 + R3​) after 1500 cycles. The equivalent circuit analysis results (Figure S7 and Table S2, Supporting Information) confirm the presence of additional electrochemical processes (R3 and CPE2) after 1500 cycles, suggesting the formation of an interfacial resistance layer at the cathode, likely caused by electrolyte oxidation. Despite these changes, similar Warburg behavior was still observed at low frequency after 1500 cycles, indicating minimal degradation of the MnHCF cathode itself.

### 2.4 SEI Layer Analyses of Mn Metal Anode

**Figure 5:**
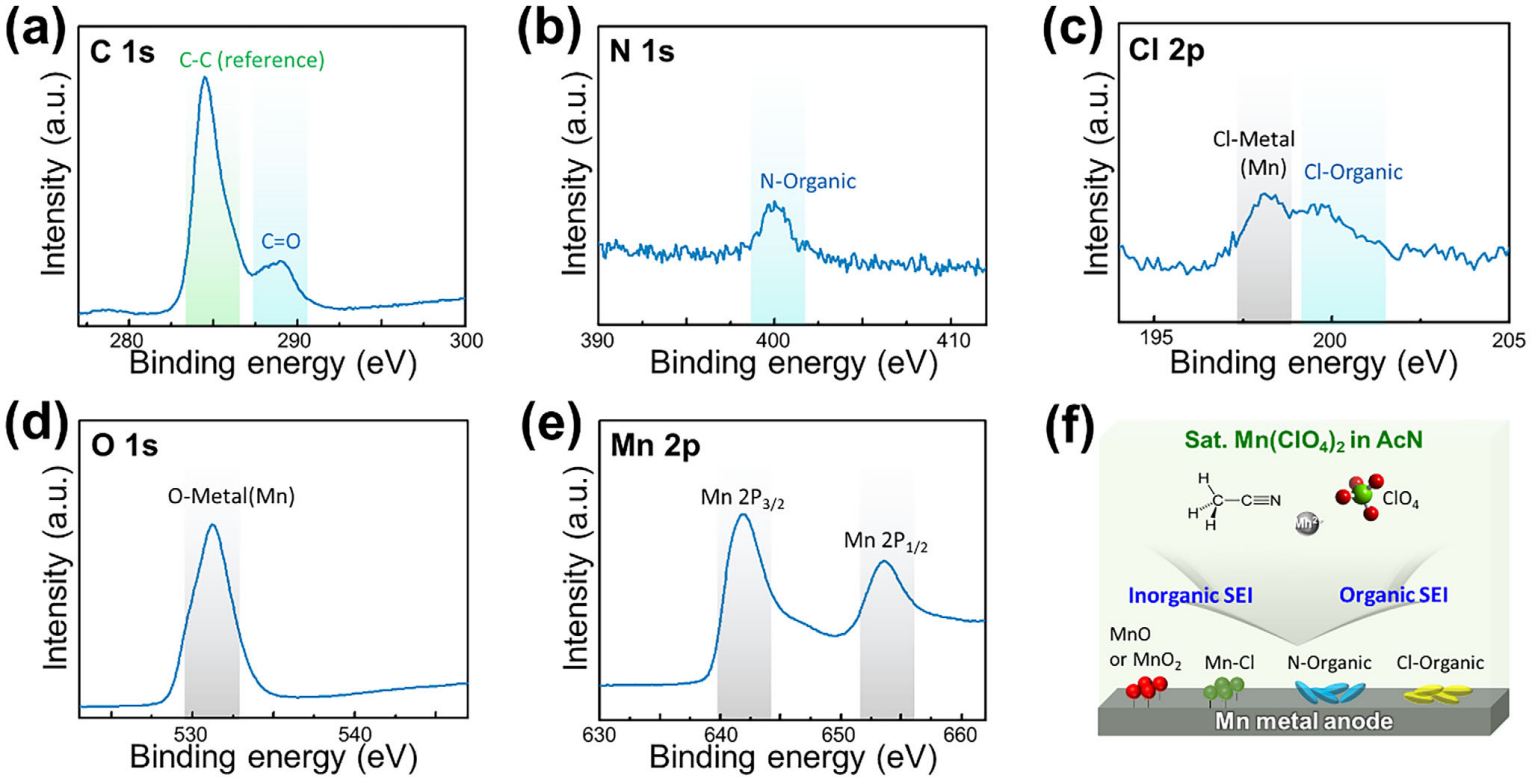
XPS spectra of Mn metal after the 1500th cycle: a) C 1s, b) N 1s, c) Cl 2p, d) O 1s, e) Mn 2p; along with a schematic illustration of the composition of the SEI layer in saturated Mn(ClO_4_)_2_/AcN electrolytes.

To further investigate the surface chemistry of the Mn metal anode after 1500 cycles, X‐ray photoelectron spectroscopy (XPS) measurements were performed, and the spectra for C 1s, N 1s, Cl 2p, O 1s, and Mn 2p are presented in **Figure** **5**. As previously discussed, manganese metal undergoes reactions during cycling, resulting in the formation of irreversible products (Figure 2). We hypothesize that Mn reacts with the saturated Mn(ClO₄)₂ in the AcN electrolyte solution, leading to its decomposition and the formation of interfacial layers on both the anode and cathode.

When analyzing the XPS data for the Mn metal anode, several significant features can be identified, shedding light on the surface chemistry after cycling. In the C 1s spectrum (Figure 5a), peaks within the 277–300 eV range are visible, with reference and additional peaks at 284 and 288 eV, respectively, corresponding to C ═ O bonds. This indicates that carbonaceous compounds from the electrolyte decompose during cycling, forming an organic SEI (solid electrolyte interphase) layer on the Mn metal anode. The N 1s spectrum (Figure 5b) shows a peak at 400 eV, attributed to N‐organic bonds, further confirming the decomposition of the AcN electrolyte.^[^ ^25^ ^]^ The Cl 2p spectrum (Figure 5c) displays two distinguishable peaks at 198 and ≈201 eV, corresponding to Cl‐metal bonds and attributed to Cl‐organic compound bonds,^[^ ^26^ ^]^ respectively. These results confirm that AcN decomposition during cycling leads to SEI formation on the Mn anode, containing organic (e.g., N‐organic, Cl‐organic) and inorganic (e.g., MnCl_x_) components.

Additionally, the O 1s and Mn 2p spectra (Figure 5d,e) indicate the presence of Mn‐O bonds, suggesting that manganese oxides (MnO_x_) are part of the SEI layer.

After 1500 cycles, the saturated Mn(ClO₄)₂ in AcN electrolyte generates a complex SEI layer comprising organic (e.g., N‐organic, Cl‐organic) and inorganic (e.g., MnO_x_, MnCl_x_) compounds on the Mn metal anode as described in Figure 5f.

The presence of a C ═ O peak in the C 1s spectrum carries significant implications, suggesting a complete collapse of the AcN electrolyte's chemical structure (CH_3_CN). The reaction of the degraded electrolyte with oxygen from the decomposition of chlorine and oxygen species from ClO_4_⁻ indicates substantial anion degradation. Furthermore, residual decomposed Cl in the electrolyte reacts with Mn metal, leading to the formation of Mn‐Cl compounds. As these compounds are insoluble in AcN organic solvents, they may contribute to the blockage of the Mn metal surface, potentially leading to performance deterioration. This SEI layer contributes to increased battery resistance and highlights the critical importance of understanding and optimizing SEI formation on Mn anodes to enhance the electrochemical performance of manganese‐ion batteries. In this field, further advancements in additive and electrolyte solution engineering are essential to develop practical organic electrolyte‐based manganese‐ion batteries.

### 2.5 Charge Storage Mechanism of MnHCF

**Figure 6:**
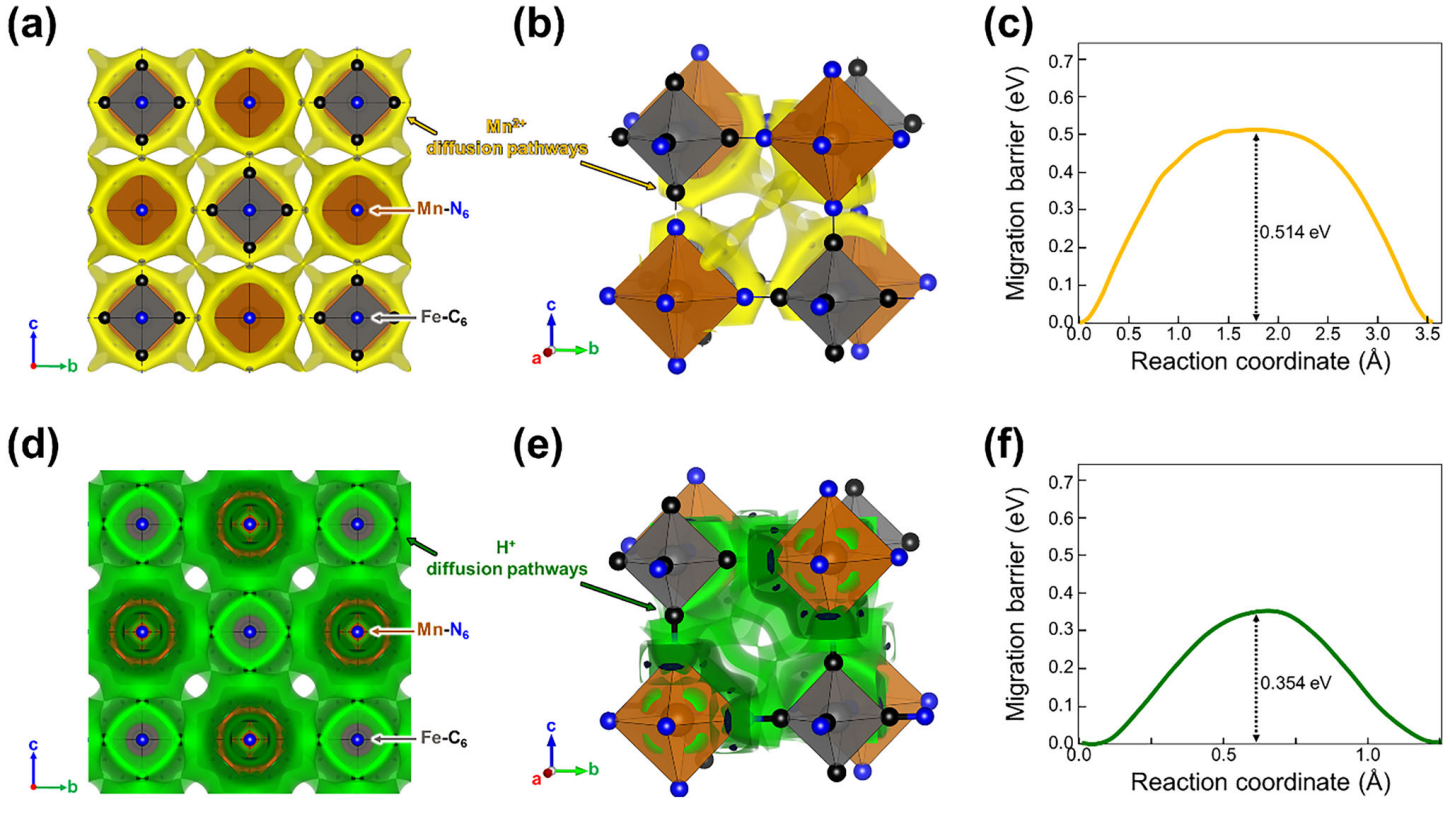
a) Mn‐ion diffusion pathway within the cubic MnHCF structure, b) localized diffusion pathways in MnHCF, c) calculated results for the Mn‐ion migration energy barrier d) proton diffusion pathway within the cubic MnHCF structure, e) localized diffusion pathways in MnHCF, f) calculated results for the proton migration energy barrier.

ICP analysis confirmed that 0.16 m of manganese was inserted into the MnHCF framework (Table S3, Supporting Information). This corresponds to a theoretical capacity of ≈51.2 mAh g^−1^, conssidering the theoretical capacity of MnHCF is 160 mAh g^−1^. However, this value differs from the observed capacity of 73.4 mAh g^−1^. This discrepancy suggests that protons from residual water in the organic solvent are also being inserted into the structure. The diffusion pathways and energy barriers of Mn^2^⁺ ion and proton within the MnHCF structure were calculated using the SoftBV program.^[^ ^27^ ^]^ As shown in **Figures** **6**a and S8 (Supporting Information), the 3D migration pathways of Mn^2^⁺ ions are visualized traversing through the FeC₆‐MnN₆ framework. Examining the local cavity (Figure 6b), the Mn^2^⁺ ions are observed to diffuse preferentially near the FeC₆ and MnN₆ sites rather than directly across the central cavity. The calculated diffusion barrier is 0.514 eV, as illustrated in Figure 6c. This relatively low energy barrier is attributed to the large diffusion channels provided by the cubic Prussian blue framework, which facilitates efficient ion transport within the structure. Furthermore, the potential incorporation of protons into the MnHCF structure was investigated by calculating diffusion pathways and energy barriers (Figure 6d). The results indicate that protons actively diffuse through the FeC₆–MnN₆ framework (Figure 6e). The internal diffusion barrier of protons within the structure was determined to be 0.354 eV, which is lower than that of manganese, suggesting that proton diffusion within the structure is energetically favorable (Figure 6f).

These results indicate that Mn^2^⁺ ion and proton can easily diffuse into the Prussian blue framework, highlighting the potential of MnHCF as a cathode material for manganese‐ion batteries. Furthermore, this suggests the viability of other transition‐metal‐based cubic Prussian blue phases as promising cathode candidates for similar applications.

**Figure 7:**
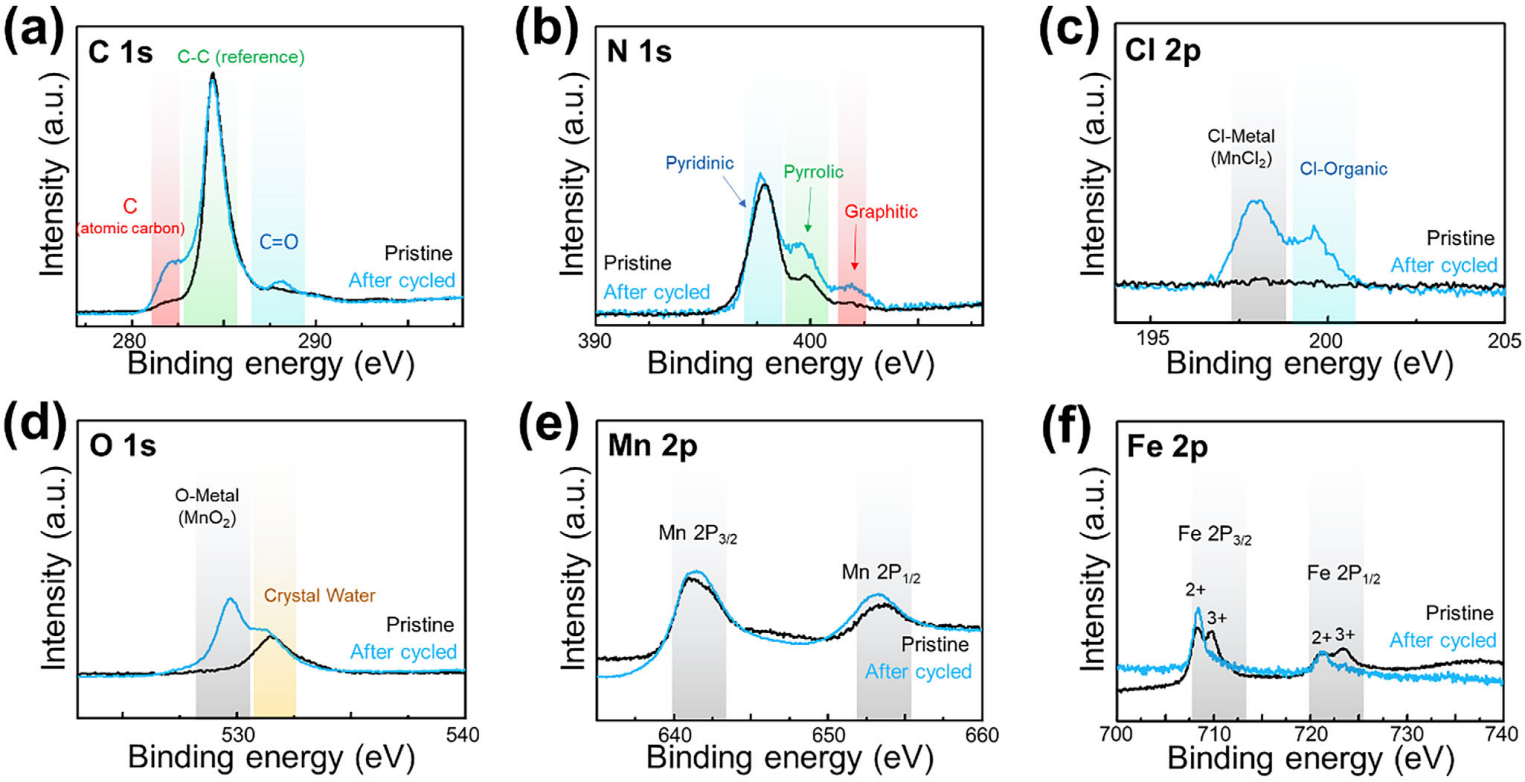
XPS spectra of MnHCF cathode, before and after the 1500th cycle: a) C 1s, b) N 1s, c) Cl 2p, d) O 1s, e) Mn 2p, f) Fe 2p.

To further investigate the cathode interface layer after 1500 cycles, XPS analyses were performed, and the spectra for C 1s, N 1s, Cl 2p, O 1s, Mn 2p, and Fe 2p are presented in **Figure** **7**. The formation of electrolyte decomposition products on the cathode surface over long‐term cycling can be attributed to the degradation of both the AcN electrolyte and the Mn(ClO_4_)_2_ salt. To evaluate the influence of C≡N signals originating from MnHCF, the obtained spectra were compared with those of pristine MnHCF.

In the C 1s spectrum (Figure 7a), peaks within the range of 277–298 eV were observed, with a reference peak at 284 eV, along with additional peaks at 282 and 288 eV, corresponding to atomic carbon and C ═ O bonds, respectively, which likely result from electrolyte decomposition. The N 1s spectrum (Figure 7b) exhibits peaks at 397.5, 399, and 402 eV. The 397.5 eV peak is attributed to C≡N bonds. Notably, when AcN decomposes at the cathode side, the intensity of peaks associated with pyrrolic‐ and graphitic‐type nitrogen increases. In contrast to the anode's SEI layer, where only pyrrolic‐type nitrogen byproducts were observed, the cathode exhibits an additional formation of graphitic‐type side products, suggesting a distinct decomposition mechanism. The Cl 2p spectrum (Figure 7c) presents two distinguishable peaks at 198 and ≈201 eV, which resemble the decomposition products observed on the anode side. Organic nitrogen‐chlorine species, Cl‐organic compounds, and insoluble MnCl_2_ deposits on the cathode surface could contribute to an increase in interfacial resistance. Additionally, the O 1s and Mn 2p spectra (Figure 7d,e) indicate the presence of manganese oxides (MnO_x_), while crystalline water remains well maintained in the pristine MnHCF structure. A comparison between pristine MnHCF and the cycled sample after 1500 cycles reveals a difference in the oxidation state of Fe. The Fe 2p spectrum (Figure 7f) demonstrates a shift in oxidation states, confirming that Fe serves as the primary redox center in the Prussian blue structure.

The formation of the cathode surface layer exhibits distinct characteristics compared to that of the anode. In particular, notable differences are observed in the decomposition of carbon and nitrogen‐containing species. Atomic carbon appears to undergo further molecular decomposition, while graphitic‐type nitrogen, which is absent on the anode, is clearly present on the cathode. These findings suggest that the anode and cathode undergo electrolyte degradation via different mechanisms, likely due to their distinct chemical potential environments.

## 3 Conclusion

Here, we successfully demonstrated the potential of organic electrolyte‐based manganese batteries. Based on organic electrolytes, addressing key challenges (hydrogen evolution) associated with aqueous systems. A MnHCF cathode and saturated Mn(ClO₄)₂ in acetonitrile (AcN) electrolyte were utilized to demonstrate reversible manganese‐ion intercalation and extraction. The MnHCF cathode exhibited an average operating voltage of 1.7 V and a discharge capacity of 73.4 mAh g^−1^ at 0.1 A g^−1^, retaining 71.1% of its capacity after 1500 cycles at 0.2 A g^−1^. Additionally, efficient 3D Mn^2^⁺ ion diffusion pathways within the MnHCF framework, with a low diffusion barrier of 0.514 eV, further validate its suitability for multivalent‐ion systems. Despite the promising electrochemical stability of the saturated Mn(ClO₄)₂ in AcN electrolyte, decomposition reactions were observed during extended cycling, leading to the formation of a complex SEI layer. XPS analysis confirmed the SEI composition, including organic (N‐organic, Cl‐organic) and inorganic (MnOx, MnCl_x_) components, contributing to increased resistance and reduced coulombic efficiency. These results highlight the importance of electrolyte engineering and SEI control in enhancing the performance and durability of manganese batteries.

This study provides critical insights into the design and optimization of organic electrolyte‐based manganese batteries, marking an important initial step toward their new application. Future efforts should focus on improving the stability and compatibility of organic electrolytes, minimizing SEI layer formation, and enhancing the overall efficiency of manganese metal deposition and dissolution by salts and electrolyte engineering. With continued advancements in cathode materials and electrolyte systems, manganese batteries can potentially become a highly competitive solution for next‐generation energy storage technologies.

## 4 Experimental Section

### Material Synthesis and Characterization of MnHCF

MnHCF was synthesized using a co‐precipitation method.^[^ ^23^ ^]^ K₃[Fe(CN)₆] and MnSO₄ solutions were mixed under 60 °C to facilitate the formation of MnHCF. The resulting precipitate was collected by centrifugation and washed several times with deionized water to remove unreacted chemicals. The purified product was then dried in an oven at 80 °C for 12 h. The morphology of the synthesized MnHCF was examined using scanning (SEM, Hitachi 8020) and transmission (TEM, FEI, Themis Z) electron microscopes equipped with energy‐dispersive X‐ray spectroscopy (EDX) for elemental analysis. Structural characterization was performed using a Rigaku Mini‐Flex 600 X‐ray diffractometer with Cu Kα radiation (λ = 1.542 Å). The crystallographic parameters of MnHCF were refined via Rietveld analysis using the GSAS.^[^ ^28^ ^]^

### Battery Test

MnHCF cathodes were prepared by mixing MnHCF powder, Super P carbon (Timcal), and polyvinylidene fluoride (Kureha Co.) binder in a weight ratio of 8:1:1. The mixture was dispersed in N‐methyl‐2‐pyrrolidone and coated onto 32 µm titanium foil (Alfa–aesar). The coated electrodes were dried at 80 °C and subsequently pressed using an electrode press to enhance adhesion and uniform thickness. For the three‐electrode cell tests, Mn metal and rod were used as an anode and the reference electrode, respectively. A glass fiber separator (Whatman) was placed between the electrodes, and a saturated Mn(ClO₄)₂ in AcN solution was used as the organic electrolyte solution. Electrochemical characterizations, including cyclic voltammetry and galvanostatic charge–discharge tests, were conducted using a Biologic VMP‐3e potentiostat/galvanostat. Impedance measurements were performed in PEIS mode over a frequency range of 0.1–200 kHz to analyze the electrochemical behavior and resistance components of the cells.

### Mn‐Ion Diffusion Path and Barrier Calculation

The diffusion pathways and energy barriers for Mn^2^⁺ ions in MnHCF structures were calculated using the softBV program.^[^ ^27^ ^]^ The diffusion characteristics were analyzed based on a structural model obtained through Rietveld refinement of powder XRD data using the GSAS software.^[^ ^28^ ^]^ 3D migration pathways of Mn^2^⁺ ions within the MnHCF framework were visualized using VESTA (version 3), providing a detailed representation of ion transport in the material.^[^ ^29^ ^]^

## Conflict of Interest

The authors declare no conflict of interest.

## Supporting information



Supporting Information

## Data Availability

The data that support the findings of this study are available from the corresponding author upon reasonable request.
