# Supplementary material for: Nonaqueous Electrolyte Rechargeable Manganese Batteries with Potassium Manganese Hexacyanoferrate Cathodes
Source: Adv Sci (Weinh). 2025 Mar 28;12(23):2500132. doi: 10.1002/advs.202500132 (PMC12199449; doi:10.1002/advs.202500132)
Supplement: Supplementary file 1 — Supporting Information [file ADVS-12-2500132-s001.docx]

Supporting Information

**Nonaqueous Electrolyte Rechargeable Manganese Batteries with Potassium Manganese Hexacyanoferrate Cathodes**

*Jangwook Pyun, Hyeonjun Lee, Seunghyeop Baek, Sangki Lee, Hyeju Kwon, Hyeongseok Lee, Chung-Yul Yoo*, Munseok S. Chae**

**
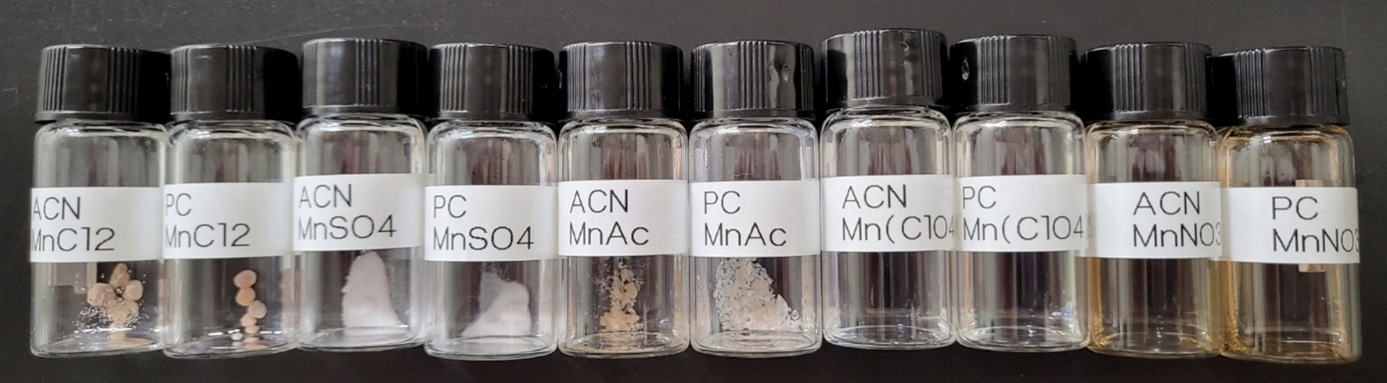
**

**Figure S1.** Images of compatibilities test between organic solution (AcN and PC) and various Mn salts (MnCl_2_, MnSO_4_, Mn acetate, Mn(ClO_4_)_2_, Mn(NO_3_)_2_

**
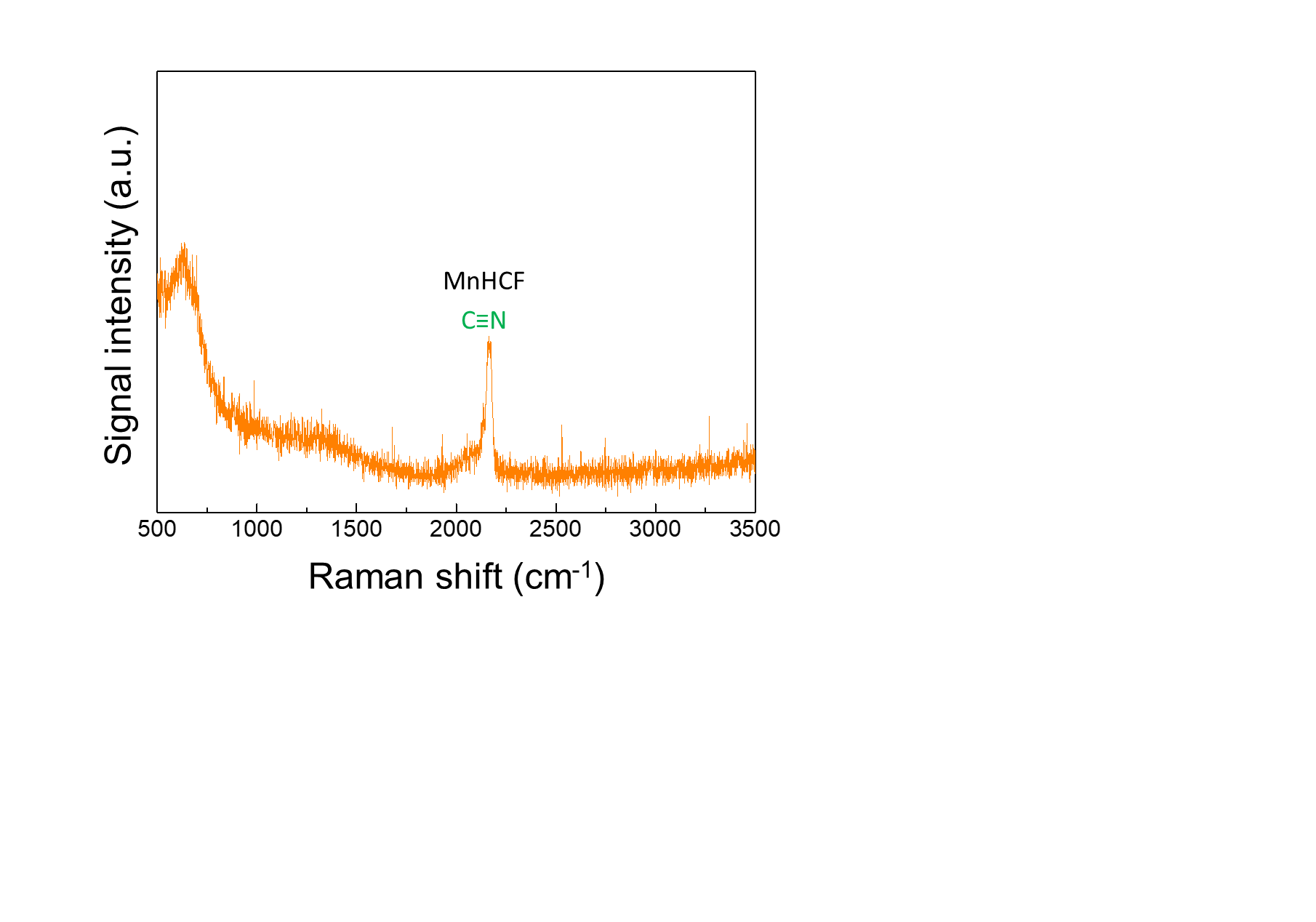
**

**Figure S2.** Raman spectra of the synthesized MnHCF powder

**Table S1.** Powder XRD Rietveld refinement results for MnHCF: atomic coordinates, site occupancies, and reliability factors at room temperature

| Crystal System  Space Group  Lattice Parameters, Volume, Z | | | | Cubic  *F m -3 m (no. 225)*  *a, b, c* = 10.491(1)  α, β, γ = 90 ^o^  V = 1154.8(2) Å^3^, *Z* = 4 | | | |
| --- | --- | --- | --- | --- | --- | --- | --- |
| Atoms | *x* | *y* | *z* | | Wyckoff | Occupancy | U_iso_ × 100 |
| Mn | 0.5000 | 0.5000 | 0.5000 | | *4b* | 1.000 | 5.9(7) |
| Fe | 0.0000 | 0.0000 | 0.0000 | | *4a* | 1.000 | 5.5(7) |
| K | 0.2500 | 0.2500 | 0.2500 | | *8c* | 0.500 | 6.8(7) |
| C | 0.0000 | 0.0000 | 0.1707(1) | | *24e* | 1.000 | 9.4(7) |
| N | 0.0000 | 0.0000 | 0.2827(1) | | *24e* | 1.000 | 8.9(7) |

*R*_p_ = [0.328](1_publ%20_pd_proc_ls_prof_R_factor), *R*_wp_ = [0.498](1_publ%20_pd_proc_ls_prof_wR_factor), *R*_exp_ = [0.530](1_publ%20_pd_proc_ls_prof_wR_expected), *R*(*F*^2^) = [0.09674](1_publ%20_refine_ls_R_Fsqd_factor), χ^2^ = 0.884

XRD: X-ray diffraction

MnHCF: manganese hexacyanoferrate

**
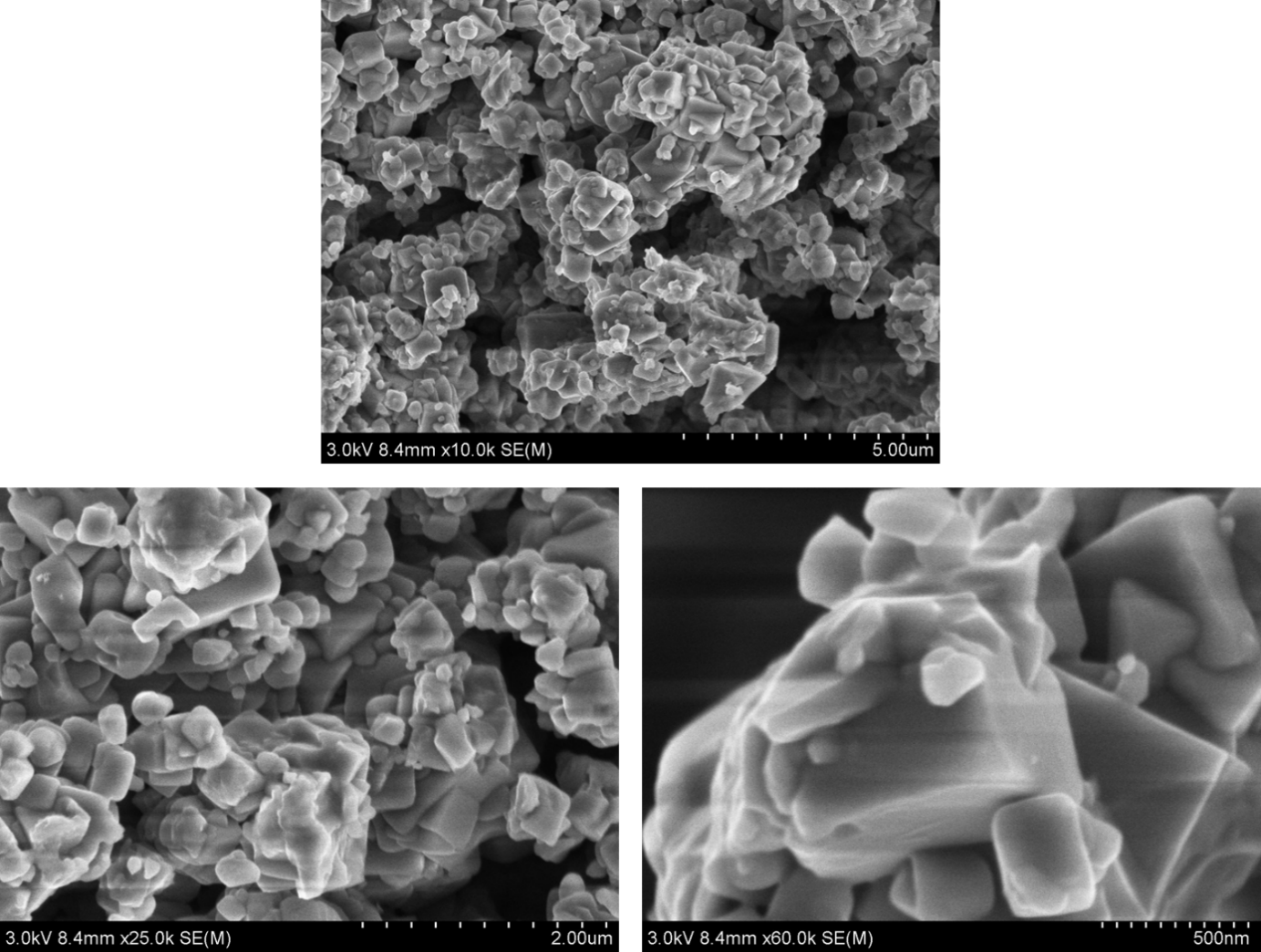
**

**Figure S3.** SEM images of synthesized MnHCF

SEM: scanning electron microscope

**
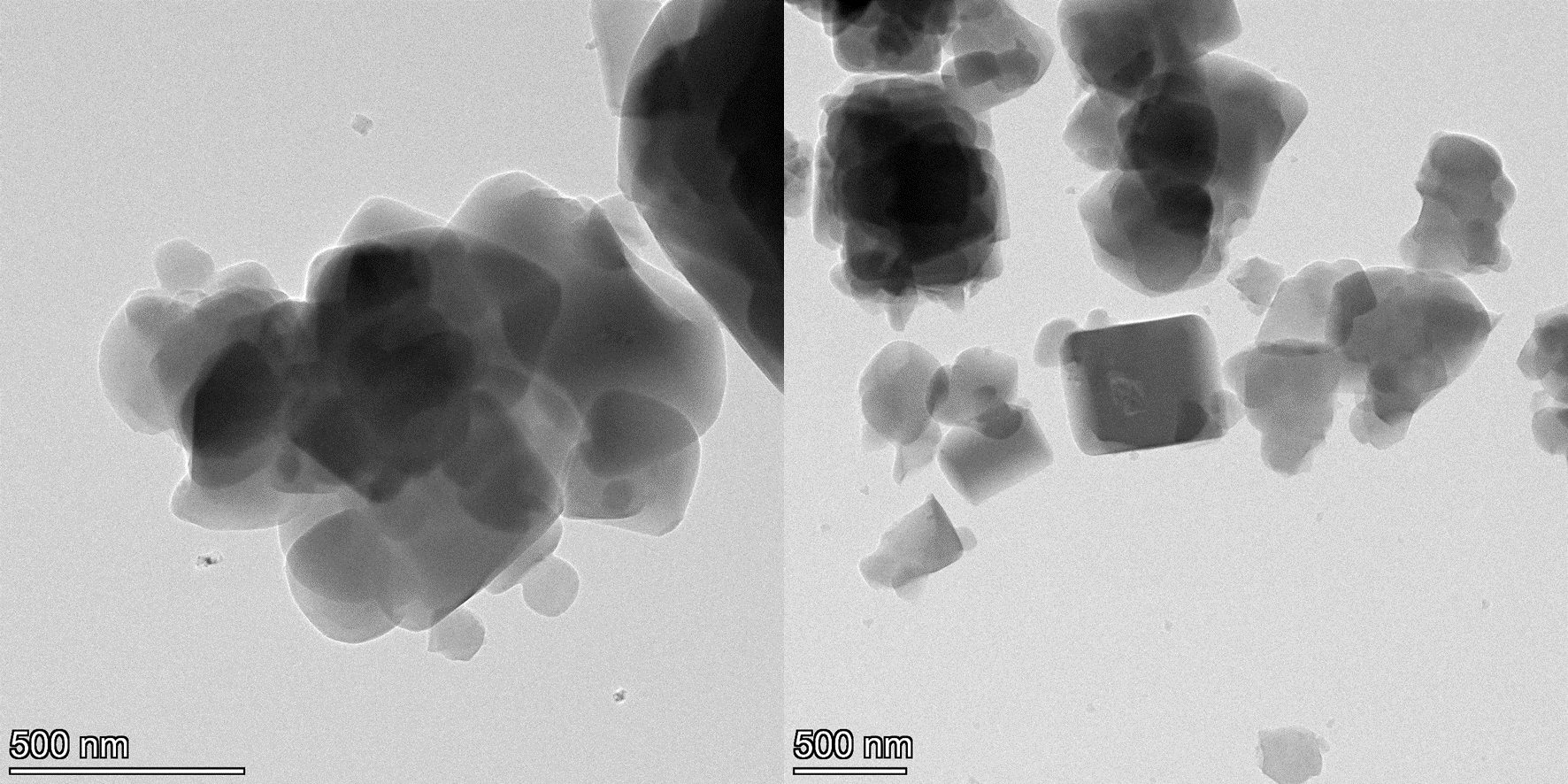
**

**Figure S4.** TEM images of synthesized MnHCF

TEM: transmission electron microscope

**
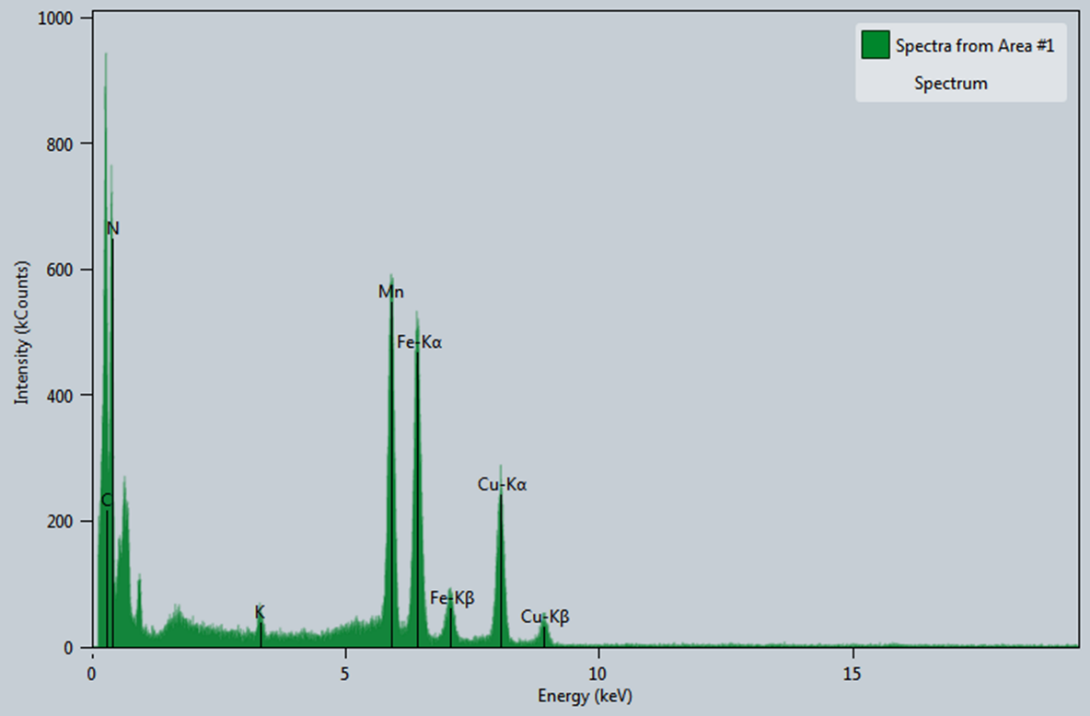
**

**Figure S5.** TEM-EDX spectra of MnHCF Powder

EDX: X-ray spectroscopy

**
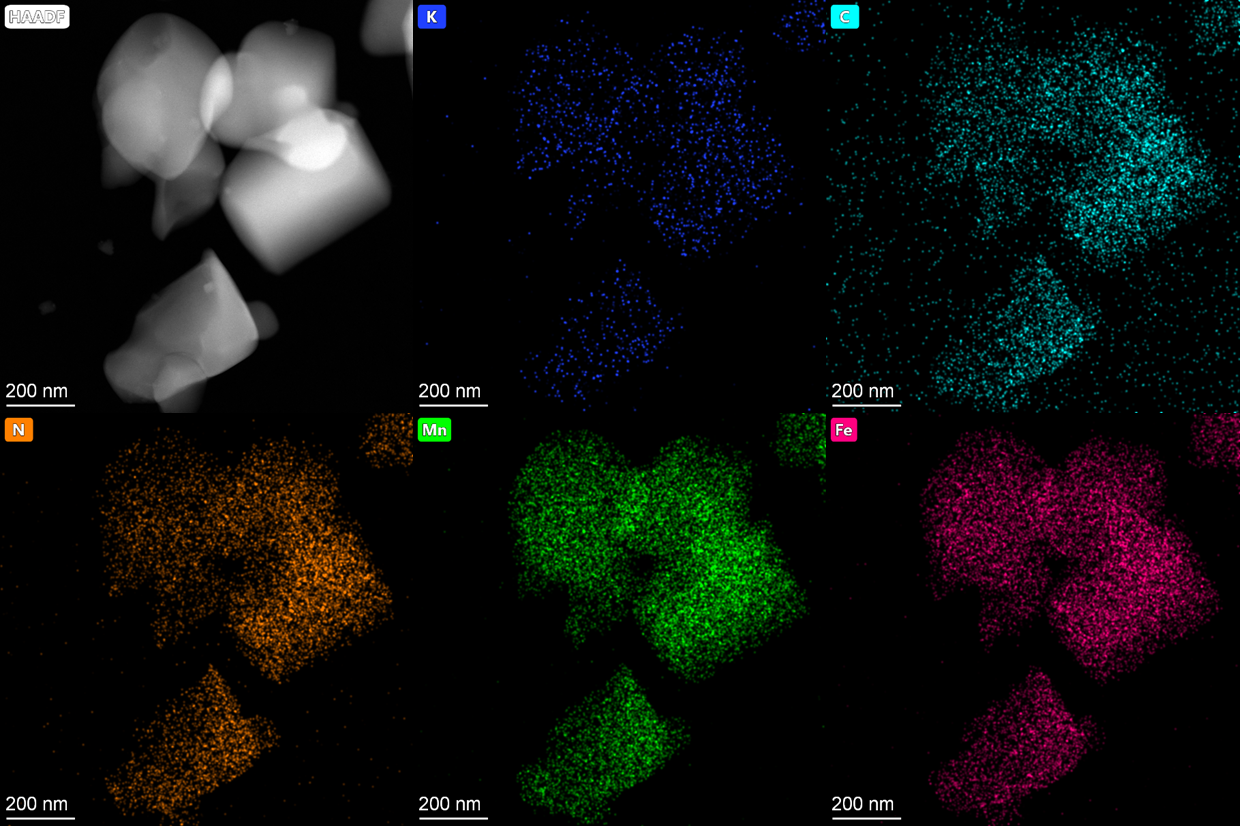
**

**Figure S6.** TEM-EDX elemental mapping of MnHCF powder


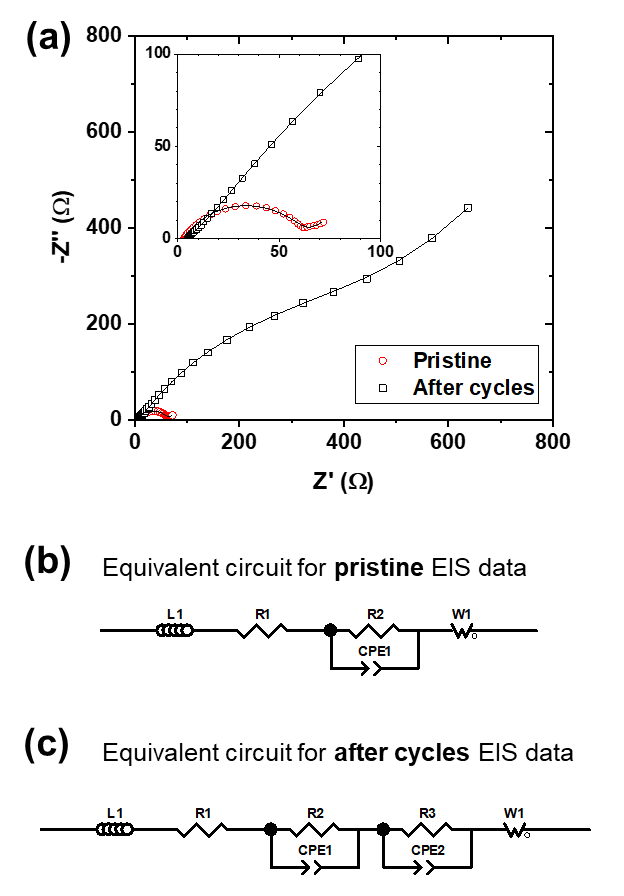


**Figure S7.** Electrochemical impedance spectroscopy analyses of pristine and after 3000 cycles : (a) Fitting data for nyquist plot, (b) equivalent circuit for pristine electrode, (c) equivalent circuit for after 3000 cycled.

**Table S2.** EIS fitting parameters of pristine and after cycles

| Pristine | | After cycles | |
| --- | --- | --- | --- |
| L (µH) | 0.978  (±0.15) | L (µH) | 0.978  (±0.44) |
| R1 (Ω) | 2.94  (±0.09) | R1 (Ω) | 4.57  (±0.47) |
| CPE1  (Ω^-1^·s^-n^) | 3.55 × 10^-4^  (±1.0 × 10^-5^) | CPE1  (Ω^-1^·s^-n^) | 7.51 × 10^-4^  (±6.1 × 10^-6^) |
| n1 | 0.67  (±0.01) | n1 | 0.66  (±0.01) |
| R2 (Ω) | 59.1  (±0.6) | R2 (Ω) | 510  (±21) |
| W1-R (Ω) | 1.63 × 10^4^  (±2.2 × 10^3^) | CPE2  (Ω^-1^·s^-n^) | 2.33 × 10^-3^  (±2.1 × 10^-4^) |
| W1-T (s) | 1.63 × 10^7^  (±1.2 × 10^6^) | n2 | 0.51  (±0.01) |
| W1-P | 0.45  (±0.02) | R3 (Ω) | 9.51  (±0.11) |
|  |  | W1-R (Ω) | 1.03 × 10^5^  (±3.5 × 10^3^) |
|  |  | W1-T (s) | 5.93 × 10^3^  (±3.1 × 10^2^) |
|  |  | W1-P | 0.67  (±0.01) |

**Table S3.** Elemental ratios estimated from ICP (Inductively coupled plasma) analysis for pristine and discharged sample.

|  | **Relative atomic ratio** | |
| --- | --- | --- |
|  | **Mn** | **Fe** |
| Pristine sample | 1.05 | 1.00 |
| Discharged sample | 1.21 | 1.00 |
| Charged sample | 1.08 | 1.00 |

**
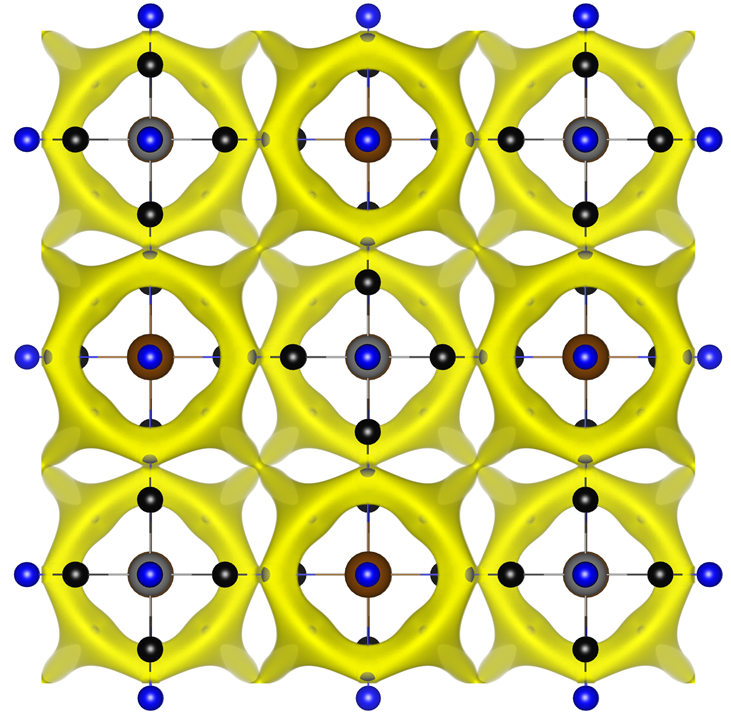
**

**Figure S8.** Mn-ion migration pathways in the cubic-MnHCF lattice
